# Work related characteristics, work-home and home-work interference and burnout among primary healthcare physicians: A gender perspective in a Serbian context

**DOI:** 10.1186/1471-2458-11-716

**Published:** 2011-09-23

**Authors:** Katarina Putnik, Inge Houkes

**Affiliations:** 1 Department of Social Medicine, Maastricht University, Research School CAPHRI, Universiteitssingel 40, P.O. Box 616, 6229 ER, Maastricht, the Netherlands

**Keywords:** Burnout, Medical doctors, Gender differences, Work-Home and Home Work Interference, Job Characteristics

## Abstract

**Background:**

Little information exists on work and stress related health of medical doctors in non-EU countries. Filling this knowledge gap is needed to uncover the needs of this target population and to provide information on comparability of health related phenomena such as burnout across countries. This study examined work related characteristics, work-home and home-work interference and burnout among Serbian primary healthcare physicians (PHPs) and compared burnout levels with other medical doctors in EU countries.

**Methods:**

Data were collected via surveys which contained Maslach Burnout Inventory and other validated instruments measuring work and home related characteristics. The sample consisted of 373 PHPs working in 12 primary healthcare centres. Data were analysed using *t-*tests and Chi square tests.

**Results:**

No gender differences were detected on mean scores of variables among Serbian physicians, who experience high levels of personal accomplishment, workload, job control and social support, medium to high levels of emotional exhaustion, medium levels of depersonalisation and work-home interference, and low levels of home-work interference. There were more women than men who experienced low job control and high depersonalisation. Serbian physicians experienced significantly higher emotional exhaustion and lower depersonalisation than physicians in some other European countries.

**Conclusions:**

To diminish excessive workload, the number of physicians working in primary healthcare centres in Serbia should be increased. Considering that differences between countries were detected on all burnout subcomponents, work-related interventions for employees should be country specific. The role of gender needs to be closely examined in future studies as well.

## Background

### Work characteristics and burnout

Work characteristics and health problems such as burnout have been extensively researched in the Western countries, including the United Kingdom, the United States of America, Scandinavian countries and the Netherlands, to mention a few. Work characteristics are defined as job demands and resources at work [1], while burnout is a syndrome characterised by emotional exhaustion (feelings of having depleted one's emotional resources), depersonalisation (distant attitude towards the work and persons at work) and decreased personal accomplishment (perceived personal ineffectiveness at work) [2]. The job demands-resources model, strongly supported by research, offers an explanation for the relationship between work characteristics and burnout [3]. Job demands, referring to aspects of work that require physical and/or psychological effort, are strongly associated with emotional exhaustion. Job resources, aspects of work that facilitate achievement of work goals and stimulate personal growth and development, are more related to depersonalization and personal accomplishment [4,5]. Job resources can buffer the negative impact of high job demands. In this study, demands are examined through workload, childcare and household duties, work-home and home-work interference, while resources are explored through variables of job control, social support and favourable work content.

Scarce knowledge exists with regards to expression of these variables in different cultural settings. One such underexplored case example is physician burnout in Serbia. We know very little about health and work characteristics of medical doctors in Serbia and the extent to which they manage to combine work and family related duties. In fact, Lešić and colleagues [6] acknowledge this lack of research and call for studies in the Serbian cultural context that would look into the work-family balance. In light of this knowledge gap and taking into consideration that burnout is one very prominent example of stress related problems of which physicians are particularly at risk [7], the present study set out to explore work characteristics, work family interference and burnout among Serbian primary healthcare physicians (PHPs), paying attention to gender and cross cultural differences.

### Serbian context

In Serbia, full-time employment and dual income families are the norm [8]. Only 1% of waged employment is part-time [9]. Home chores and childcare are still largely in women's hands, regardless of labour participation, education level or occupation [10]. Instead of obtaining help from the partner, Serbian women can be expected to receive help from a mother or a paid helper [11]. Thus, despite greater female labour emancipation, traditional values still prevail in the home sphere.

Regarding the health sector, there are 116 primary healthcare centres (PHC), where 31% of all doctors work [12]. Unlike in many other countries, there are more female (64%) than male physicians (36%). Among general practitioners (GPs), the gender disparity is even more pronounced, with 95% female representation [13]. To our knowledge, only two small-scale studies on Serbian medical doctors working in primary care have been conducted. One study was conducted on 39 GPs [14] and the second study [6] among 38 GPs. These studies are a valuable contribution to the initial assessment of physicians' health in Serbia. However, studies with larger samples and more variables under examination are needed for a more comprehensive overview of prevalence of burnout. Furthermore, a comparison of burnout levels in Serbia with other international research is needed in order to position the findings of this specific cultural setting in relation to other European countries.

### Perspective on gender, work characteristics, work-home interference and burnout

When examining work and health conditions of employees, including gender in the analysis is of paramount importance in order to provide a better understanding of men's and women's conditions of work and illness aetiology.

Numerous evidence points to the connection between work circumstances and employees' health. For example, speed of work has increased in most countries since the last decade of the previous century [15], and this work intensity is related to negative health outcomes, such as increased stress [16]. Amount and quality of social contacts, along with the job autonomy are also relevant variables to examine. Adequate social support and high levels of work autonomy are related to more positive health outcomes [3]. Besides work characteristics, the interplay between the work and home sphere is also important to consider as this is related to stress and burnout [17]. In this study work-home interference (WHI) is defined as the extent to which work related tasks impede on fulfilment of home duties [18], while home-work interference (HWI) refers to interference of home roles with performance of work duties [19]. Gender differences regarding the role of WHI and HWI are still not clear. In Western populations, some studies found greater work-home and home-work interference for females [20,21], while others found similar effects for both genders [22,23].

Both men and women can go on to develop stress, and subsequently burnout, in situations where work characteristics and WHI are unfavourable [17]. The relationship between gender and burnout is not yet clear [24]. In some studies gender differences on the overall level of burnout were not detected [7], while some gender differences in burnout subcomponents were found [7,25,26].

In this study, we focus on gender differences in the prevalence of work characteristics, work-home and home-work interference and burnout among Serbian physicians working in PHCs, and compare burnout findings with other international results. Given that this is an early explorative study of the situation in Serbia, more complex gender analyses and interrelationships with health outcomes will not be addressed here.

To achieve the aim of the study, we will answer the following research questions:

1. What is the level and prevalence of work characteristics among Serbian PHPs and are there any gender differences?

2. What is the level and prevalence of home and childcare responsibilities, work-home and home-work interference among Serbian PHPs and are there any gender differences?

3. What is the level and prevalence of burnout among Serbian PHPs and are there any gender differences?

4. What is the level of physicians' burnout in comparison to other European countries, that is, the Netherlands, Hungary and Italy?

## Methods

### Sample

A cross-sectional study based on a survey was conducted during the months of July and August 2008. Self-reported anonymous questionnaires were distributed by directors or head nurses to 850 doctors. To increase interest in participation in the study, personal contact was established with the directors and/or head nurses of the PHCs. The study was explained, and they were offered to receive a report with the findings and recommendations for practice, upon the end of the data collection. In this study, 12 PHCs (8 from Belgrade and 4 from other cities) took part. The mix of small and large PHCs was equally represented in Belgrade and other cities. All doctors working in the PHC were eligible to take part in the study. Excluded from participation were doctors who were on sick leave or holidays during the data collection period (approximately three weeks per institution). The response rate was 44%, that is, 373 respondents answered the questionnaire. All the participants were informed in writing that their participation was voluntary and that data provided were treated confidentially. By completing the survey, the physicians gave their consent to participate in the study. According to the Dutch law (Wet Medisch-Wetenschappelijk Onderzoek met Mensen/Medical Research Involving Human Subjects Act) our type of study based on paper and pencil survey does not require ethical committee approval.

Sample reflected gender differences in PHCs in Serbia: 84% were female and 16% were male. Mean age for both men and women was 47 (*SD (men) *= 10.15; *SD (women) *= 8.48). Of the total sample, 31% of physicians were aged 20-44 and the rest were in the age group 45-64, reflecting a greater proportion of older participants in this study. Most respondents (77%) had children; 61% of them lived with children and spouse or partner, 8% lived alone, 10% with the partner, 7% with children and 14% in some other form of family constitution. On average, participants had between 1 and 2 children ($\bar{x}$ = 1.4 children), the average age of children was 19 years. Proportion of the sample with younger children, defined in this study as younger than 12 years of age, was 23%. Years of working experience were similar between men (19.8 years) and women (19.6 years). The working hours and structure of work did not vary among the participants.

### Measurements

The scales for workload, job control, work content, social support and work-home and home-work interference were translated from Dutch to Serbian using the back translation method. Following this, refinements were made using more iterative processes to check that the meaning stayed the same in both languages, a procedure suggested by American Educational Research Association, American Psychological Association and National Council on Measurement in Education [27].

I. Workload was measured by an eight item scale (α = .87) based on the Job Autonomy Questionnaire [28]. The participants indicated their agreement with each item on a four-point scale (1 = never, 4 = very often).

II. Job control was measured by four items (α = .72) of the Inventory of feelings of motivation and demotivation [29]_. _Responses ranged from 1 = not at all to 4 = very much.

III. Work content was assessed via five items based on the Job Diagnostic Survey [30] and developed by Janssen and colleagues [31], which examines creativity, independence and chances for feedback on person's work. The scale (α = .82) offered four answering options (1 = never, 4 = very often).

IV. Social support was measured by three five item scales deducted from the Questionnaire on Organizational Stress-Doetinchem (VOS-D), each assessing the social support received from supervisor/colleague, partner and family/friends [32]. The scores on three subscales were averaged to yield a sum score. Reliability of the scale was good (α = .82). Items were rated on four point scales (1 = never, 4 = always).

V. Home and childcare responsibilities were each measured by a one item measure created for the purpose of this study: "What is the percentage of the household duties carried out by yourself?" and "What is the percentage of the childcare duties carried out by yourself?"

VI. Work-home and home-work interference were assessed via two scales consisting of 13 items [18,33]. Work-home interference scale assesses the amount of interference from work to home domain, such as work clashing with home and family plans. It consists of seven items (α = .90). Home-work interference examines the extent of interference from home to work domain, for example having difficulties concentrating at work because of being preoccupied with domestic matters. The scale contains six items (α = .84). The respondents answered via five point scale (1 = never, 5 = always).

VII. Burnout was measured using the Serbian translated version of the Maslach Burnout Inventory-General Survey [14], which contained 22 items and three scales. Emotional exhaustion was measured by ten items (α = .89), depersonalisation by five items (α = .68) and reduced personal accomplishment by seven items (α = .75). Previous research shows that the reliability of depersonalisation score is usually low [5,34]. Responses were made on a six-point scale (0 = never, 6 = every day).

### Data analyses

We started with the preliminary analyses (Means, SD and Pearson correlations for the subgroups of males and females). Since no Serbian national cut-off points exist for different levels of burnout, we used the cut-off points from the Utrechtse Burnout Schaal (UBOS) manual [35]. No standardised cut-off points for work characteristics were found, so we used general rules of thumb, which indicate when high levels of certain variable are experienced. According to Houkes [36], for scales ranging from 1-4, the cut-off point according to the rule of thumb is 2.5, and for scales 1-5, the cut-off point is 3.5. For example, workload scale ranges between 1 (never) and 4 (very often), and individuals who score above 2.5, experience 'often' or 'very often' high workload. In order to examine gender differences in mean values of variables and determine whether the prevalence differs among men and women independent samples *t*-tests and chi-square tests were performed, respectively. To examine burnout differences between Serbian physicians and their colleagues in European countries, findings were compared to the studies of physician burnout in Hungary, the Netherlands and Italy. All studies used MBI as a measure of burnout. Studies in the Netherlands [7] and Italy [26] were conducted on samples of general practitioners (GPs), while the Hungarian study [25] included physicians from various specialties and GPs. To examine differences between Serbian physicians and their colleagues in other countries, one sample *t*-tests were carried out. All the analyses were performed using SPSS 15.01 version.

## Results

### Research question 1: Work characteristics among Serbian PHPs

Mean levels of work characteristics, as well as gender differences and Pearson correlations are reported in Table 1.

**Table 1:** Descriptive characteristics, gender differences and correlations between variables for males in the top right corner (n = 58) and females in the bottom left corner (n = 315)

Variable (range)		Men		Women		Gender difference			Variable											
		***Mean***	***SD***	***Mean***	***SD***	***t***	***df***	***p***	**1**	**2**	**3**	**4**	**5**	**6**	**7**	**8**	**9**	**10**	**11**	**12**

1	Age	47.43	10.16	47.38	8.48	.04	72.53	.97	1	-.20	-.20	.15	.17	.15	.06	.19	-.19	-.03	-.36**	.01
2	Workload (1-4)	2.83	.58	2.93	.60	-.17	370	.24	-.05	1	-.35**	-.29*	-.04	.64**	.38**	-.26	.06	.48**	-.04	.02
3	Job control (1-4)	2.89	.76	2.74	.66	1.5	37	.12	-.02	-.09	1	.38**	-.28*	-.52**	-.35**	-.17	-.16	-.36**	-.18	.24
4	Work content (1-4)	2.89	.73	2.92	.58	-.34	70.94	.73	.14*	-.03	.33**	1	-.20	-.33*	-.29*	-.05	-.21	-.47**	-.35**	.48**
5	Lack of social support (1-4)	1.66	.37	1.74	.40	-1.55	358	.12	.10	.15**	-.30**	-.34**	1	.27*	.58**	-.05	-.03	.38**	.40**	-.21
6	Work on home interference (1-5)	2.42	.85	2.58	.80	-1.35	371	.18	-.02	.54**	-.16**	-.11	.26**	1	.72**	-.03	.10	.51**	.12	-.09
7	Home on work interference (1-5)	1.65	.58	1.68	.61	-.44	370	.66	-.07	.27**	-.13*	-.17**	.30**	.57**	1	.00	.20	.47**	.26	-.26*
8	Childcare provided by yourself (%)	43.37	22.02	63.84	20.63	-5.76	282	.00	.21**	.05	-.12	-.10	.20**	.02	.02	1	.54**	-.23	-.21	.06
9	Household duties carried out by yourself (%)	51.72	28.03	69.99	23.00	-5.16	357	.00	.05	.06	-.11	-.04	.04	.04	.03	.60**	1	.01	-.01	-.23
10	Emotional exhaustion(0-6)	2.25	1.33	2.51	1.30	-1.42	368	.16	-.01	.63**	-.20**	-.16**	.25**	.57**	.31**	.03	.06	1	.28*	-.15
11	Depersonalisation (0-6)	.65	.65	.75	.92	-.75	368	.45	-.20**	.31**	-.05	-.17**	.15**	.32**	.35**	-.08	-.11*	.46**	1	-.45**
12	Personal accomplishment (0-6)	5.10	1.06	5.19	.81	-.63	70.07	.53	.04	-.06	.18**	.26**	-.18**	-.04	-.21**	-.09	.07	-.12*	-.21**	1

** *p *≤ .01 level (2-tailed); * *p *≤ .05 level (2-tailed). Missing values were handled by pairwise deletion.

We found that Serbian physicians had mostly favourable working conditions. No gender differences on mean levels of work characteristics were detected (Table 1). Physicians reported overall high levels of job control ($\bar{x}$_(men) _= 2.89 and $\bar{x}$_(women) _= 2.74), work content ($\bar{x}$_(men) _= 2.89 and $\bar{x}$_(women) _= 2.92) and low levels of lack of social support $\bar{x}$_(men) _= 1.66 and $\bar{x}$_(women) _= 1.74).

We also looked at the prevalence of these variables and examined gender differences. These results are reported in Table 2. We found that significantly more women suffered from low control at work (42.4%) compared to men, where prevalence was 24.1%. Both genders experienced high workload: 73.6% of women and 65.5% of men complained of high amount of work. The prevalence of unfavourable work content and lack of social support was small. This was reflected by the finding that more than 70% of the sample experienced high possibility for creativity in their work and felt they had a lot of chances to learn new things and develop as measured by work content. Physicians experienced high levels of support from their colleagues and supervisors, which was reflected in the finding that only 2% of physicians experienced low social support.

**Table 2:** Gender differences in work characteristics, work-home interference, home-work interference and burnout prevalence

Variable	Men	Women	Gender differences between levels of variables		
	Percent (%)	Percent (%)	***χ***^**2**^	*df*	*p*
Workload^a^					
Low^b ^(≤2.5)	34.5	26.4	1.58	1	.21
High (> 2.5)	65.5	73.6			
Job control^a^					
Low (≤2.5)	24.1	42.4	**6.80**	1	**.01**
High (> 2.5)	75.9	57.6			
Work content^a^					
Low (≤2.5)	27.6	23.2	.51	1	.48
High (> 2.5)	72.4	76.8			
Lack of social support^a^					
Low (≤2.5)	93.1	97.7	.31	1	.58
High (> 3.5)	3.4	2.3			
Work-home interference^a^					
Low (≤3.5)	89.7	86.7	.39	1	.53
High (> 3.5)	10.3	13.3			
Home-work interference^a^					
Low (≤3.5)	98.3	99	.27	1	.60
High (> 3.5)	1.7	1			
Emotional exhaustion^b^					
Low (0-1.12)	22.4	17.0	1.55	2	.46
Medium (1.13-2.49)	36.2	33.7			
High (> 2.50)	41.4	49.4			
Depersonalisation^b^					
Low (0-.59)	48.3	55.4	**7.72**	2	**.02**
Medium (.60-1.59)	46.6	30.1			
High (> 1.60)	5.2	14.4			
Personal accomplishment^b^					
Low (0-3.70)	10.3	4.2	3.85	2	.15
Medium (3.71-4.70)	15.5	17.3			
High (> 4.71)	74.1	78.5			

Bold font indicates significant results

^a ^Cut off points based on Houkes (2002) [36]

^b ^Cut off points based on Schaufeli & van Dierendonk (2000) [35]

### Research question 2: Home and childcare responsibilities and work-home and home-work interference

Levels of home and childcare responsibilities, work-home and home-work interference and gender differences are reported in Table 1. The prevalence of these variables, and any associated gender differences in prevalence are reported in Table 2.

Analyses revealed that home and childcare responsibilities differed between men and women. Women in our sample carried out disproportionally more childcare than men (63.84% versus 43.37%), and more home chores than men (69.99% versus 51.71%).

Regarding the balance of work and home duties, we found that WHI was higher than HWI for men and women (Table 1). However, no significant gender differences were identified between mean levels of WHI and HWI.

Concerning the prevalence of WHI, we saw that only 13.3% of female physicians and 10.3% of male physicians experienced high WHI (Table 2). Prevalence of high HWI was 1% among women, and 1.7% among men.

Since the presence of young children involves more childcare duties, and can thus be associated with higher WHI and HWI, we carried out further analyses to examine this (results not reported in tables). Contrary to previous findings, the presence of younger children aged less than 12 years was not found to be related to WHI. In fact, having children older than age 12 produced slightly higher levels of WHI for both women and men. Women with older children experienced mean level of WHI of 2.71, while women with younger children experienced mean level of WHI of 2.61, *t*(239) = .85, *p *≥ .05. Men with older children had a mean level of WHI of 2.55, while men with younger children experienced the level of WHI of 2.24, *t*(40) = .99, *p *≥ .05. We also found the same pattern of higher HWI for physicians with children older than 12, and again the difference between the two groups was non significant Women with older children had a mean score of 1.83 of HWI, while women with younger children had a mean score of 1.67 of HWI, *t*(238) = 1.71, *p *≥ .05. Men with older children had a mean score of 1.73 of HWI, while men with younger children had a mean score of 1.44, *t*(30.44) = -1.95, *p *≥ .05.

### Research question 3: Burnout prevalence

Levels of burnout are reported in Table 1 and its prevalence in Table 2. Based on the cut-off points [35], emotional exhaustion among Serbian PHPs was medium to high, depersonalisation was medium and personal accomplishment was high. We also examined the prevalence of emotional exhaustion, depersonalisation and personal accomplishment. 49% of women and 41% of men experienced high levels of emotional exhaustion. Medium level of depersonalisation was experienced by both men and women. Unlike in other studies, in our sample, significantly more women experienced high levels of depersonalisation than men. 14.4% of women compared to 5, 2% of men experienced high depersonalisation, χ^2^(2) = 7.72, *p* = .02. Feelings of personal accomplishment at work were high for both men and women, with 78.5% of women, and 74.1% of men reporting high levels of personal accomplishment. No statistically significant gender differences were detected, but there was a tendency for greater proportion of women to experience low personal accomplishment than men (10% vs. 4%).

### Research question 4: European comparison of burnout levels among physicians

The findings on mean levels of physicians' burnout in the current study, Hungary, the Netherlands and Italy are reported in Table 3.

**Table 3:** Cross-country comparison on mean levels of burnout among physicians

	**Current study GPs** ***(Mean)***	**Hungary**^**a**^ ***(Mean)***	***t***	***df***	***p***	**The Netherlands**^**b**^ ***(Mean)***	***t***	***df***	***p***	**Italy**^**c**^ ***(Mean)***	***t***	***df***	***p***
			
Emotional exhaustion	2.47	1.88	8.68	369	.00	2.06	6.03	369	.00	1.85	9.12	369	.00
Depersonalisation	0.73	1.02	-6.30	369	.00	1.71	-21.37	369	.00	1.22	-10.67	369	.00
Personal accomplishment	5.17	5.07	2.35	369	.02	5.08	2.12	369	.03	5.50	-7.32	369	.00

^a ^Adam et al. (2008). [25] In the study n = 420

^b ^Twellaar et al. (2008) [5] In the study n = 349

^c ^Grassi and Magnani (2000) [26] In the study n = 182

When comparing the current study with other European research carried out on similar samples in Hungary [25], the Netherlands [7] and Italy [26], it appears that Serbian physicians experience the highest levels of emotional exhaustion. Depersonalisation levels among Serbian physicians are significantly lower than in other countries (*p *= .000). Personal accomplishment is high among physicians in all countries, but differences between Serbian physicians and physicians in other countries have been detected. Level of personal accomplishment is significantly higher among Italian than Serbian physicians, (*t*(369) = -7.32, *p *= .00), but lower among Hungarian (*t*(369) = 2.35, *p *= .02) and Dutch physicians (*t*(369) = 2.12, *p *= .03).

To summarise, the cross-country results indicate that the current sample of Serbian physicians experienced the highest level of emotional exhaustion and lowest level of distancing from patients (depersonalisation) when compared to other samples.

## Discussion

Our results show that physicians in Serbia have favourable work characteristics in terms of high job control, favourable work content and social support, but face adversely high work demands. The indication of high job demands may not be surprising, considering that in Serbia, there are 281 primary healthcare physicians per 100, 000 people [12]. As a comparison, countries, such as Hungary, Italy and the Netherlands, have much higher numbers of physicians per 100, 000 inhabitants: 316, 606 and 329 respectively [37].

Women physicians in our sample carry out more childcare and household duties than their male colleagues, confirming previous findings that female emancipation in Serbia has not yet translated fully into the home sphere [10,38].

In our study, levels of WHI were higher than levels of HWI. However, currently, WHI does not seem to be problematic; perhaps, a buffering factor against high WHI/HWI is the organization of the working time schedule. In Serbia, employees of PHCs do not have night duties and work either mornings or afternoons. Since offices are shared between physicians of different shifts, this prevents structural overtime. Unlike in some other countries, like the Netherlands, on-call duties are also not part of PHPs' work. Predictability is associated with lower stress levels [39], and such stable working hours can be one potential explanatory factor for the finding that physicians with younger children do not experience more WHI than their colleagues with older children.

Lack of gender differences with respect to the levels of WHI/HWI is in line with the findings of Winants and colleagues [40]. In our sample such a lack of difference may be surprising, since women carry out higher proportion of home and childcare duties than the males in this study. One possible explanation might lie in the extensive web of social network that women can rely on, which ensures that the childrearing duties do not spill over to work. It may also be due to a traditional gender ideology that prevails in Serbia. According to Greenstein, women with traditional gender ideology do not perceive unequal division of household labour as unfair, and it does not lead to increased tension between work and family sphere since the unequal division of chores is consistent with their gender ideology [41].

We found job demands to be high among our sample of participants. Based on job demands-resources model [3], high workload can explain high exhaustion. The more workload physicians experience, the higher the pace of their work and the more effort they exert, leading to higher exhaustion. Considering that they work directly with patients, this can be an emotionally demanding encounter, creating emotional exhaustion. However, high levels of exhaustion experienced among Serbian physicians may also be a reflection of demands stemming from wider economic and political situation. For many years, economic instability has created financial worries about having enough to provide for the family's needs. As an illustration, average salary of the medical doctor in 1999 in Serbia was €85 per month [42]. There was also a lot of political instability and changes in governments. The international community imposed sanctions in the 1990s as well as travel restrictions until 2010. These political events, together with the cultural norm of caring for the elderly family members, may have created increased strain on persons. This strain can manifest itself in the form of higher emotional exhaustion. Cross-cultural comparison lends support to this argument. Physicians in Serbia had the highest level of emotional exhaustion: higher than their Hungarian, Dutch or Italian colleagues.

Depersonalisation levels among Serbian physicians were lower than those of their colleagues in other countries. This indicates that Serbian physicians show less emotional distance (depersonalisation) from patients than Hungarian, Dutch or Italian physicians. Such a finding may be a reflection of the specific cultural context. In Serbia, unlike in the Netherlands for example, it is less socially acceptable to have an emotional distance from the patients. However, whether in reality physicians are truly that involved with the clients is worth exploring further, since social desirability might have played a role in the findings.

Unlike in other studies [43,44], women in our sample reported higher levels of distancing to patients (depersonalisation) than men. An explanation for such finding may be related to different coping and communication styles between men and women. Men's greater employment of avoidance coping style [45], may mean that it protects them from being overly involved with the patients. Such over-involvement with the patients is seen as a stage preceding development of depersonalisation [5]. Thus, men's avoidance coping style may act as a buffer against distancing to patients (depersonalisation). Feminine communication style characterized by greater involvement with the clients, may at a certain point become too strenuous, resulting in depersonalisation, which acts as a form of coping mechanism [46].

Prevalence of personal accomplishment is generally high in international studies, indicating that physicians feel efficient in their work [7,14,25]. High level of professional accomplishment might be inherent in the profession of general physicians, since they often see clients who generally recover fast following the therapy prescribed. In cases where more serious diagnoses are found, physicians refer these patients to secondary and tertiary levels of care.

Our findings show no gender differences on mean scores of work characteristics. However, gender differences were noted regarding higher percentage of women than men having low control, high levels of depersonalisation, and greater tendency for being represented in the low personal accomplishment group. This finding indicates that women may be a particularly vulnerable group. Future research could explore in more depth the underlying mechanisms and variables associated with such poorer work and health characteristics of women.

The main drawback of our study is that no available data exist for burnout cut-off points in Serbia. As we utilized cut-off points standardized on a Dutch sample, the interpretation regarding the level of burnout should be done with caution because a different threshold for burnout might exist in the Serbian population. Scholars suggest using only nation-specific cut-off points [47]. Considering that these were unavailable, we believe it is better to have some cut-off points as a potential indicator of severity of the problem, rather than none at all. Another limitation of the study is that it was based on cross-sectional data, so no conclusion about causality can be drawn.

### Recommendations for future research

Our study shows that mean values for studying gender differences may not be sensitive enough to uncover complexities of gender expressions in relation to issues of work and health, which were detectable when examining levels of work characteristics, work-home duties or burnout components. It is being recommended that in future studies, it might be worth exploring the similarity or difference in mechanisms that operate in producing particular outcomes for men and women. This might provide a better understanding of gendered expressions of health [48]. Furthermore, we suggest a more fine tuned approach to gender research in the future.

Our final remark concerns the need for research on the topic of work and health in different cultural settings. Considering that national differences were found on all burnout subcomponents, we call for further and more in-depth examination of work and health related issues across countries.

### Recommendations for policy and practice

We also formulate several main recommendations for policy and practice. First, given high workload, the number of physicians working in Serbian PHCs should be increased to help the physicians deal with the excessive work demands. Second, the position of female physicians in the workplace should be considered, given the finding that more women than men feel low control and high depersonalisation at work. More empowerment, trust and positive feedback may need to be communicated to these female employees. Finally, we make a call for culturally sensitive interventions, given the finding that all burnout subcomponents differ between Serbia and other countries. For example, targeting emotional exhaustion among Serbian physicians should be a priority, while among Dutch and Italian GPs depersonalisation is a more important symptom of burnout to be addressed. Furthermore, specific cultural norms and values may produce variant health outcomes across countries and insights on such differences/similarities can provide useful directions for proper implementation of work and health recommendations in different settings.

## Conclusion

We conclude that Serbian PHPs in this study experienced unfavourable conditions in terms of high workload and medium to high emotional exhaustion. They had a medium level of depersonalisation (which was lower than in other international findings), but unlike many other studies, women tended to be more represented in the high depersonalisation group than men. Level of personal accomplishment was high among men and women, and across the countries. We saw however, inter-country differences on all burnout subcomponents, indicating that the role of nation-specific, cultural aspects might be important to consider in the future research.

## List of abbreviations used

GP: General practitioner; PHC: Primary healthcare centre; PHP: Primary healthcare physician; WHI: Work-home interference; HWI: Home-work interference

## Declaration of competing interests

The authors declare that they have no competing interests.

## Authors' contributions

KP and IH were responsible for the design of the study and this paper. KP carried out data collection and statistical analyses. KP and IH drafted and revised the manuscript and gave approval for the final version of the manuscript to be published.

## Pre-publication history

The pre-publication history for this paper can be accessed here:

http://www.biomedcentral.com/1471-2458/11/716/prepub
